# Dasatinib inhibits PD-L1 expression via a proteasomal pathway in pancreatic ductal adenocarcinoma cells

**DOI:** 10.7150/jca.124262

**Published:** 2025-11-05

**Authors:** Ching-Chung Ko, Hui-Ying Li, Pei-Ming Yang

**Affiliations:** 1 Department of Medical Imaging, Chi Mei Medical Center, Tainan 71004, Taiwan.; 2 Department of Health and Nutrition, Chia Nan University of Pharmacy and Science, Tainan 71710, Taiwan.; 3 School of Medicine, College of Medicine, National Sun Yat-Sen University, Kaohsiung 80424, Taiwan.; 4 Graduate Institute of Cancer Biology and Drug Discovery, College of Medical Science and Technology, Taipei Medical University, Taipei 11031, Taiwan; 5 PhD Program for Cancer Molecular Biology and Drug Discovery, College of Medical Science and Technology, Taipei Medical University and Academia Sinica, Taipei 11031, Taiwan.; 6 TMU Research Center of Cancer Translational Medicine, Taipei 11031, Taiwan.; 7 Cancer Center, Wan Fang Hospital, Taipei Medical University, Taipei 11696, Taiwan.; 8 Taipei Medical University (TMU) and Affiliated Hospitals Pancreatic Cancer Groups, Taipei Cancer Center, Taipei Medical University, Taipei 11031, Taiwan.

**Keywords:** pancreatic ductal adenocarcinoma, dasatinib, immune checkpoint inhibitor, PD-L1

## Abstract

Pancreatic ductal adenocarcinoma (PDAC) is an aggressive malignancy with a 5-year survival rate of below 8%. Standard chemotherapy regimens, including gemcitabine and FOLFIRINOX (fluorouracil, leucovorin, irinotecan, and oxaliplatin), offer limited clinical benefits. Although immune checkpoint inhibitors (ICIs) have revolutionized cancer immunotherapy, PDAC remains largely unresponsive to ICI monotherapy. In this study, we demonstrate that dasatinib, a multi-targeted tyrosine kinase inhibitor, reduces programmed death ligand 1 (PD-L1) expression in PDAC cells via a proteasome-dependent degradation pathway. Moreover, PD-L1 levels were correlated with dasatinib sensitivity, suggesting its utility as a predictive biomarker. These findings not only elucidate a novel mechanism of dasatinib's action but also provide a strong rationale for combining dasatinib with ICIs to overcome immune resistance and enhance therapeutic efficacy against PDAC.

## Introduction

Pancreatic cancer (PC) is among the deadliest malignancies, with a 5-year survival rate of below 5% and a median survival of just 6 months when surgical resection is not feasible 1. Approximately 80%-90% of cases arise as pancreatic ductal adenocarcinoma (PDAC) from the exocrine pancreas, and its nonspecific early symptoms make timely diagnosis difficult with current screening methods 2,3. PDAC's intrinsic resistance to both radiotherapy and standard chemotherapies, including gemcitabine, nanoparticle albumin-bound paclitaxel, and the FOLFIRINOX (fluorouracil, leucovorin, irinotecan, and oxaliplatin) regimen, yields only modest extensions in survival 4-9. Immune checkpoint inhibitors (ICIs) targeting cytotoxic T lymphocyte-associated antigen 4 (CTLA-4), programmed death 1 (PD-1), and PD ligand 1 (PD-L1) have revolutionized many cancers but have largely failed in PDAC, which exhibits primary resistance to monotherapy 10,11. Only the rare 1%-2% of PDAC cases with a mismatched repair deficiency (dMMR) or high microsatellite instability (MSI-H), which correlates with a higher tumor mutation burden and a more-inflamed microenvironment, respond to pembrolizumab as a second-line treatment 12-16. Unraveling the molecular drivers of lesion development, carcinogenesis, and treatment resistance, and finding ways to reprogram the tumor microenvironment toward a T-cell-inflamed state are critical for devising novel, more-effective therapies.

SRC, a non-receptor protein tyrosine kinase, plays a critical role in cellular signaling pathways, regulating key biological functions such as cell growth, differentiation, adhesion, and migration. In PDAC, aberrant SRC activation significantly contributes to disease progression 17. Dasatinib, an ATP-competitive tyrosine kinase inhibitor (TKI) originally developed to target both SRC and ABL kinases, also inhibits KIT, ephrin receptors, and various other kinases 18,19. Although dasatinib is approved by the US Food and Drug Administration (FDA) for treating Philadelphia chromosome-positive (Ph+) chronic myeloid leukemia (CML) and acute lymphoblastic leukemia (ALL) 20, phase II clinical trials have demonstrated that dasatinib, whether used alone or in combination with gemcitabine or FOLFOX (fluorouracil, leucovorin, and oxaliplatin), does not provide significant clinical benefits for advanced PDAC patients 21-23. Nevertheless, its tolerability in patients suggests potential for further investigation. Identifying predictive biomarkers and exploring combination therapies could improve the efficacy of dasatinib in PDAC treatment.

In this study, we identified a significant correlation between dasatinib sensitivity and PD-L1 expression in PDAC cells, with higher PD-L1 levels associated with increased sensitivity to the drug. In contrast, dasatinib sensitivity was not correlated with SRC expression in these cells. Furthermore, our results indicate that dasatinib inhibits PD-L1 expression via a proteasome-dependent pathway. These findings provide novel insights into the mechanisms underlying dasatinib's action in PDAC and could inform future therapeutic strategies.

## Materials and Methods

### Bioinformatics analyses of published data

Drug sensitivity and gene expression data from cancer cell lines, obtained from the Cancer Therapeutics Response Portal (CTRP 24-26) project, were retrieved from the DepMap database (https://depmap.org/portal/) 27. Correlations between drug sensitivity and gene expressions were analyzed using Pearson's correlation.

### Materials

Dulbecco's modified Eagle medium (DMEM; #11965084), Roswell Park Memorial Institute (RPMI)-1640 (#22400071), L-glutamine (#25030081), non-essential amino acids (NEAA; #11140050), sodium pyruvate (#11360070), and an antibiotic-antimycotic solution (#15240062) were obtained from Gibco (Grand Island, NY, USA). Fetal bovine serum (FBS; #35-010-CV) was obtained from Corning (Tewksbury, MA, USA). A GENEzol TriRNA Pure Kit (#GZX100) was obtained from Geneaid (New Taipei City, Taiwan). IQ2 SYBR Green Fast qPCR System Master Mix (#DBU-006) was obtained from Bio-Genesis Technologies (Taipei, Taiwan). An iScript cDNA Synthesis Kit (#1708891) was obtained from Bio-Rad Laboratories (Hercules, CA, USA). Dasatinib (#D-3307) was purchased from LC Laboratories (Woburn, MA, USA). Dimethyl sulfoxide (DMSO; #D5879), chloroquine (#C6628), phosphatase inhibitor cocktail tablets (#04906837001), and protease inhibitor cocktail tablets (#11873580001) were obtained from Sigma-Aldrich (St. Louis, MO, USA). MG132 (#A11043) was purchased from Adooq BioScience (Irvine, CA, USA). 3-(4,5-Dimethylthiazol-2-yl)-2,5-diphenyl tetrazolium bromide (MTT; #AF-L11939) was obtained from Alfa Aesar (Ward Hill, MA, USA). Radioimmunoprecipitation assay (RIPA) lysis and extraction buffer (#89901) was purchased from Thermo Fisher Scientific (Waltham, MA, USA). Bradford protein assay (#5000006), dual-color protein marker (#1610374), 10× sodium dodecyl sulfate (SDS)-glycine running buffer (#1610772), Trans-Blot Turbo RTA mini 0.2-µm nitrocellulose transfer kit (#1704270), and other reagents for the Western blot analysis were purchased from Bio-Rad Laboratories. PD-L1 (#GTX104763), SRC (#GTX50504), phospho-Tyr416-SRC (#GTX134837), and GAPDH (#GTX100118) antibodies were obtained from GeneTex (Hsinchu, Taiwan). Interferon regulatory factor 1 (IRF1) (#8478) and phospho-Tyr705-signal transduction and activator of transcription 3 (STAT3) (#9145) antibodies were obtained from Cell Signaling Technology (Beverly, MA, USA). The STAT3 (#sc-482) antibody was obtained from Santa Cruz Biotechnology (Santa Cruz, CA, USA). The LC3B (#18725-1-AP) antibody was obtained from Proteintech Group (Rosemont, IL, USA). Horseradish peroxidase (HRP)-conjugated anti-mouse (#115-035-003) and anti-rabbit (#111-035-003) secondary antibodies were purchased from Jackson Laboratory (Bar Harbor, MA, USA). An enhanced chemiluminescence (ECL) reagent (#NEL105001EA) was purchased from PerkinElmer (Waltham, MA, USA).

### Cell culture

AsPC-1 (#60494). BxPC-3 (#60283), HPAC (#60495), and PANC-1 (#60284) cells were obtained from the Bioresource Collection and Research Center (BCRC; Hsinchu, Taiwan). These cells were cultured in DMEM (HPAC and PANC-1) or RPMI-1640 (AsPC-1 and BxPC-3) supplemented with 10% FBS, 1 mM sodium pyruvate, a 1% NEAAs, 2 mM L-glutamine, and 1% antibiotic-antimycotic solution. They were grown in a humidified 37 °C, 5% CO_2_ incubator.

### Cell-viability assay

Cells were plated in a 96-well plate and allowed to adhere overnight. The following day, the medium was replaced with 200 μL of drug-containing medium in each well. After 72 h of incubation, 50 μL of an MTT solution (2 mg/mL) was directly added to the wells, and cells were incubated for an additional 4 h. Subsequently, the medium was removed, and the resulting MTT formazan crystals were dissolved in 200 μL of DMSO. Absorbance was measured at 570 and 650 nm on a microplate reader (Bio-Tek Instruments, Winooski, VT, USA). Cell viability was determined by subtracting the absorbance at 650 nm from that at 570 nm and normalizing the values to untreated control cells.

### Real-time quantitative polymerase chain reaction (qPCR)

Total RNA was extracted using the GENEzol TriRNA Pure Kit. First-strand cDNA was synthesized with the iScript cDNA Synthesis Kit. PCR amplification was performed using IQ2 SYBR Green Fast qPCR System Master Mix on a QuantStudio1 Real-Time PCR System (Thermo Fisher Scientific). Primer sequences used were as follows: PD-L1: forward 5′-TATGGTGGTGCCGACTACAA-3′ and reverse 5′-TGCTTGTCCAGATGACTTCG-3′; and β-actin: forward 5′-GTTGCTATCCAGGCTGTGCT-3′ and reverse 5′-AGGGCATACCCCTCGTAGAT-3′.

### Western blot analysis

Cells were rinsed twice with ice-cold phosphate-buffered saline (PBS) and centrifuged at 1,500 rpm for 5 min. The cell pellet was then resuspended in RIPA lysis buffer and incubated on ice for 30 min, with vortexing every 5 to 10 min. The lysate was subsequently centrifuged at 16,000 ×*g* and 4 °C for 20 min, and the supernatant was collected. The protein concentration was measured using the Bradford protein assay. Equal amounts of protein were mixed with loading buffer and subjected to SDS-polyacrylamide gel electrophoresis (SDS-PAGE). Following electrophoretic separation, proteins were transferred onto a nitrocellulose membrane. The membrane was blocked with 5% non-fat milk for 30 min and incubated overnight at 4 °C with primary antibodies. The next day, the membrane was treated with an HRP-conjugated anti-rabbit or anti-mouse secondary antibody for 2 h. Proteins were detected using an ECL reagent, and signals were visualized with the GE Amersham Imager 600 (GE Healthcare Life Sciences, Marlborough, MA, USA).

## Results and Discussion

### Correlation of dasatinib sensitivity with PD-L1 expression in PDAC cells

**Figure 1:**
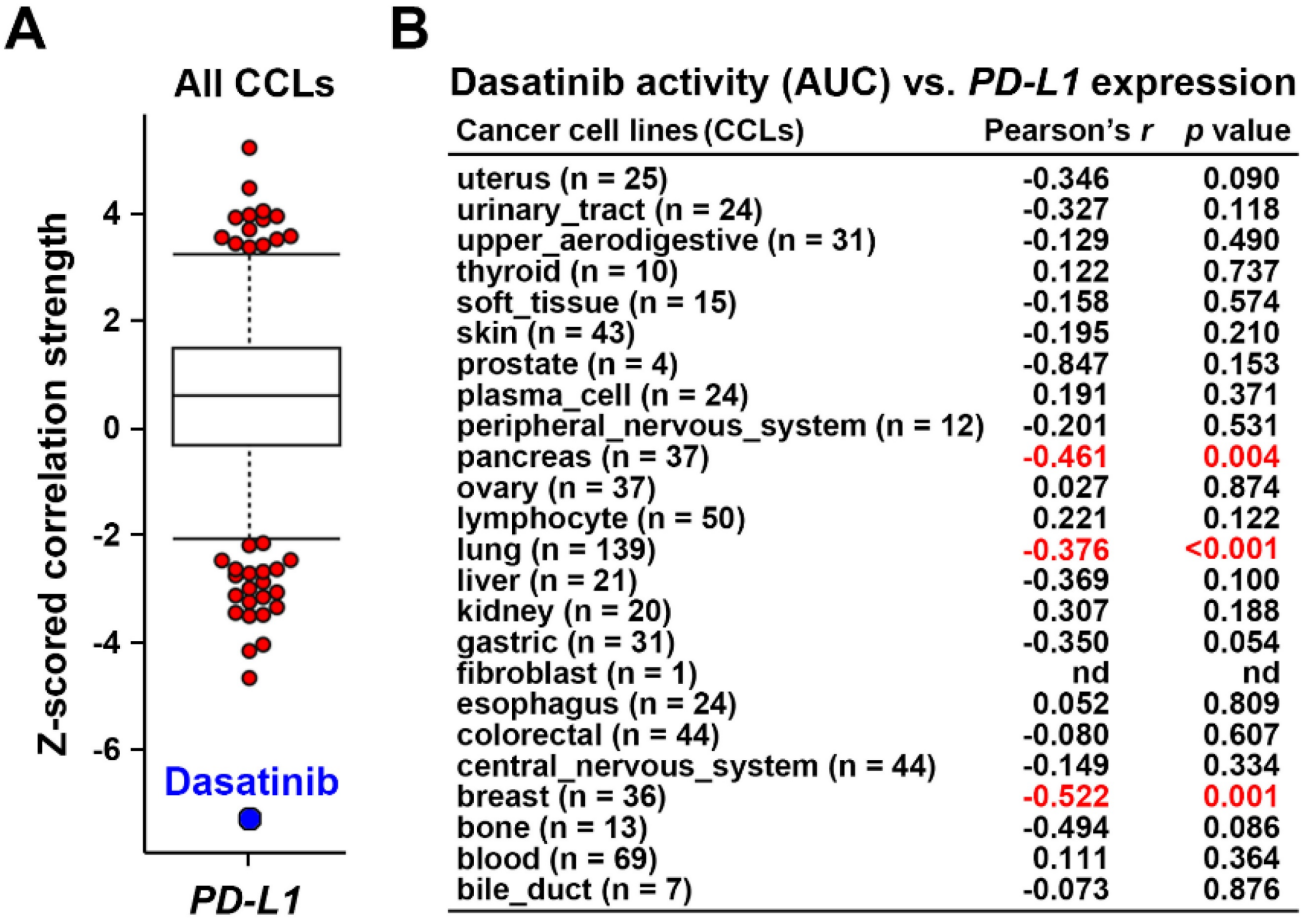
** Dasatinib drug sensitivity correlates with PD-L1 expression.** (**A**) Drug response profiles correlated with *PD-L1* mRNA expression in cancer cell lines (CCLs) were analyzed using the CTRP database. (**B**) The correlation between dasatinib drug activity (area under curve, AUC) and *PD-L1* mRNA expression in each CCL type is shown.

**Figure 2:**
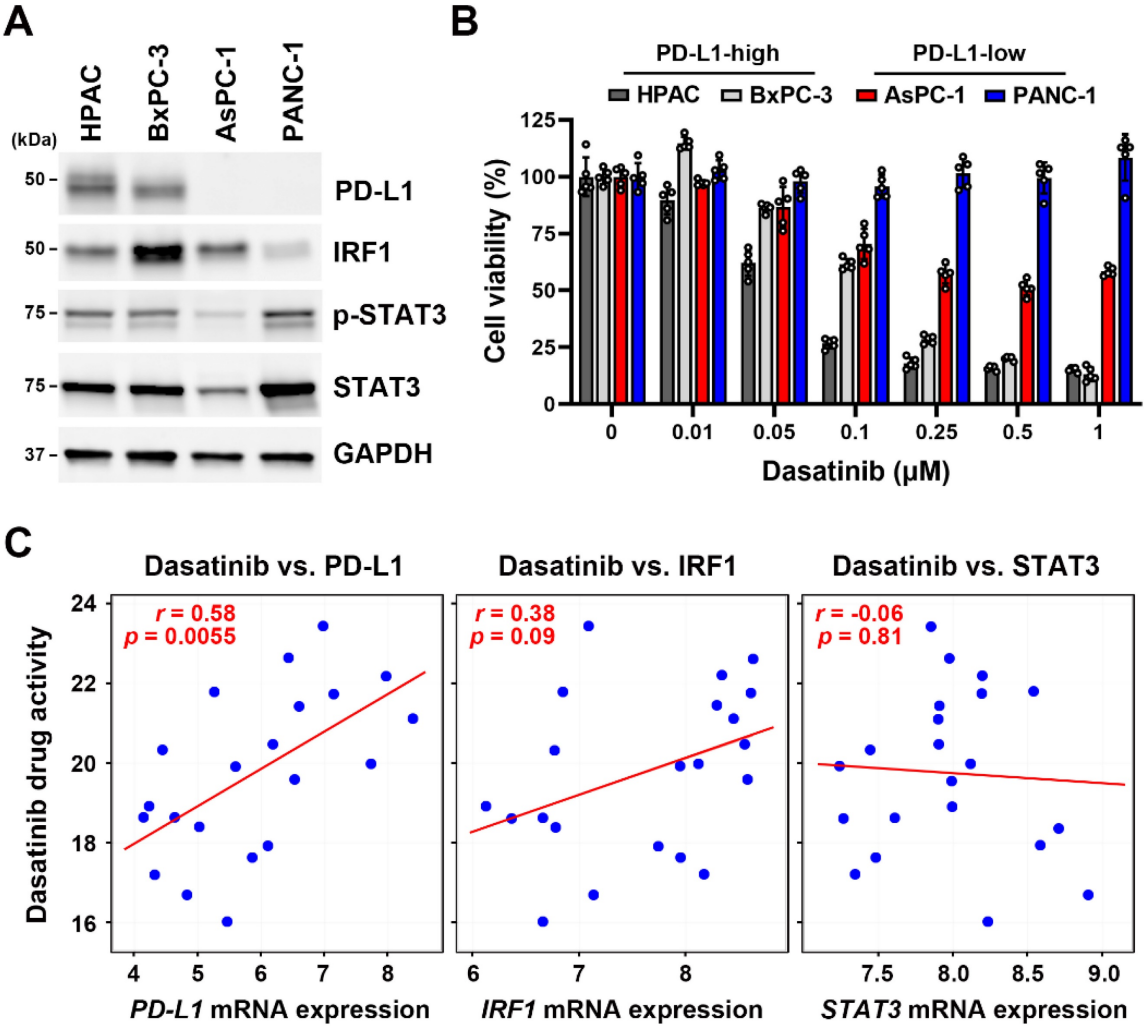
** Dasatinib exhibits higher anticancer activity in PDAC cells that highly express PD-L1.** (**A**) Protein expressions of PD-L1, IRF1, p-STAT3, and STAT3 in HPAC, BxPC-3, AsPC-1, and PANC-1 cells were compared using a Western blot analysis. (**B**) HPAC, BxPC-3, AsPC-1, and PANC-1 cells were treated with various concentrations of dasatinib for 72 h. Cell viability was determined by an MTT assay. (**C**) Scatter plots show correlations of dasatinib drug activity with *PD-L1*, *IRF1*, and *STAT3* mRNA expressions in PDAC cell lines.

Using the Cancer Therapeutics Response Portal (CTRP) database 24-26 via the CellMinerCDB website (https://discover.nci.nih.gov/rsconnect/cellminercdb/) 28, we found that cancer cells with higher *PD-L1* messenger (m)RNA expression were more responsive to dasatinib (**Fig. 1A**), especially in pancreatic, lung, and breast cancers (**Fig. 1B**). To validate this correlation, four PDAC cell lines (HPAC, BxPC-3, AsPC-1, and PANC-1) were examined. HPAC and BxPC-3 cells, which have higher PD-L1 expression, demonstrated greater sensitivity to dasatinib compared to PD-L1-negative AsPC-1 and PANC-1 cells (**Fig. 2A**,**B**). While PD-L1 expression is known to be regulated by STAT3/IRF1 signaling 29, no correlation was found of STAT3 or IRF1 protein levels with PD-L1 expression in the four PDAC cell lines (**Fig. 2A**). Additionally, *STAT3* and *IRF1* mRNA levels were not linked to dasatinib sensitivity (**Fig. 2C**). These findings suggest that PD-L1 may serve as a biomarker for dasatinib's effectiveness in treating PDAC.

### Dasatinib sensitivity was not correlated with SRC in PDAC cells

**Figure 3:**
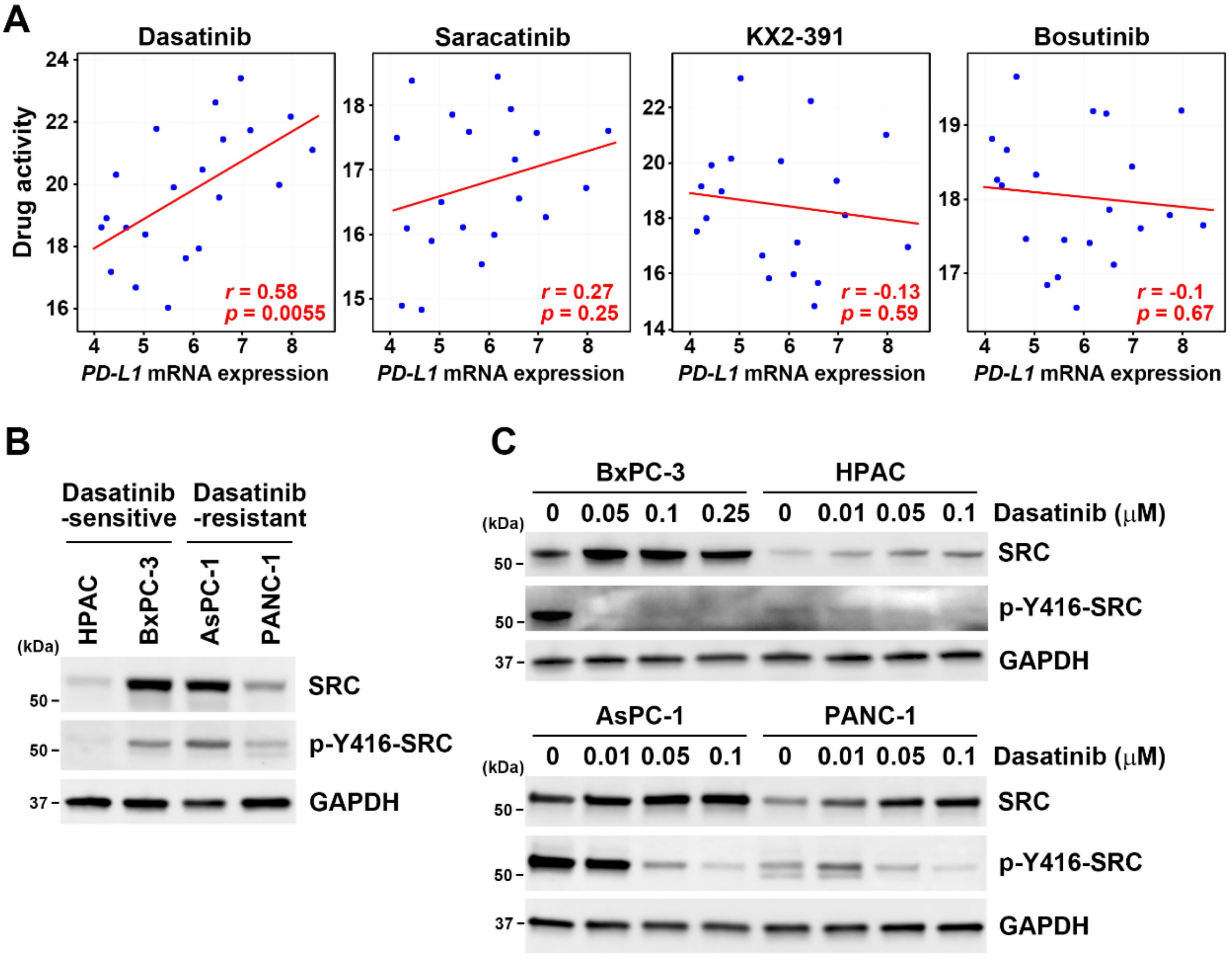
** Dasatinib drug sensitivity was not correlated with SRC expression.** (**A**) Scatter plots show correlations between drug activity of SRC inhibitors (dasatinib, saracatinib, KX2-391, and bosutinib) and *PD-L1* mRNA expression in PDAC cell lines. (**B**) Protein expressions of p-Y416-SRC and SRC in HPAC, BxPC-3, AsPC-1, and PANC-1 cells were compared using a Western blot analysis. (**C**) HPAC, BxPC-3, AsPC-1, and PANC-1 cells were treated with the indicated concentrations of dasatinib for 24 h. A Western blot analysis was performed to determine expressions of the p-Y416-SRC and SRC proteins.

The CTRP data analysis revealed that unlike dasatinib, activity of other related TKIs were not associated with *PD-L1* mRNA expression (**Table 1**). Representative scatter plots for SRC inhibitors, including dasatinib, saracatinib, KX2-391, and bosutinib, are presented in **Fig. 3A**. Additionally, neither the active (Tyr416-phosphorylated) nor total SRC protein levels (**Fig. 3B**) were correlated with dasatinib sensitivity in the four PDAC cell lines tested (**Fig. 2B**). Moreover, dasatinib effectively inhibited SRC activity in these cells (**Fig. 3C**), from which we concluded that dasatinib sensitivity in PDAC cells is not linked to SRC inhibition.

### Dasatinib inhibited PD-L1 expression via a proteasome-dependent pathway

**Figure 4:**
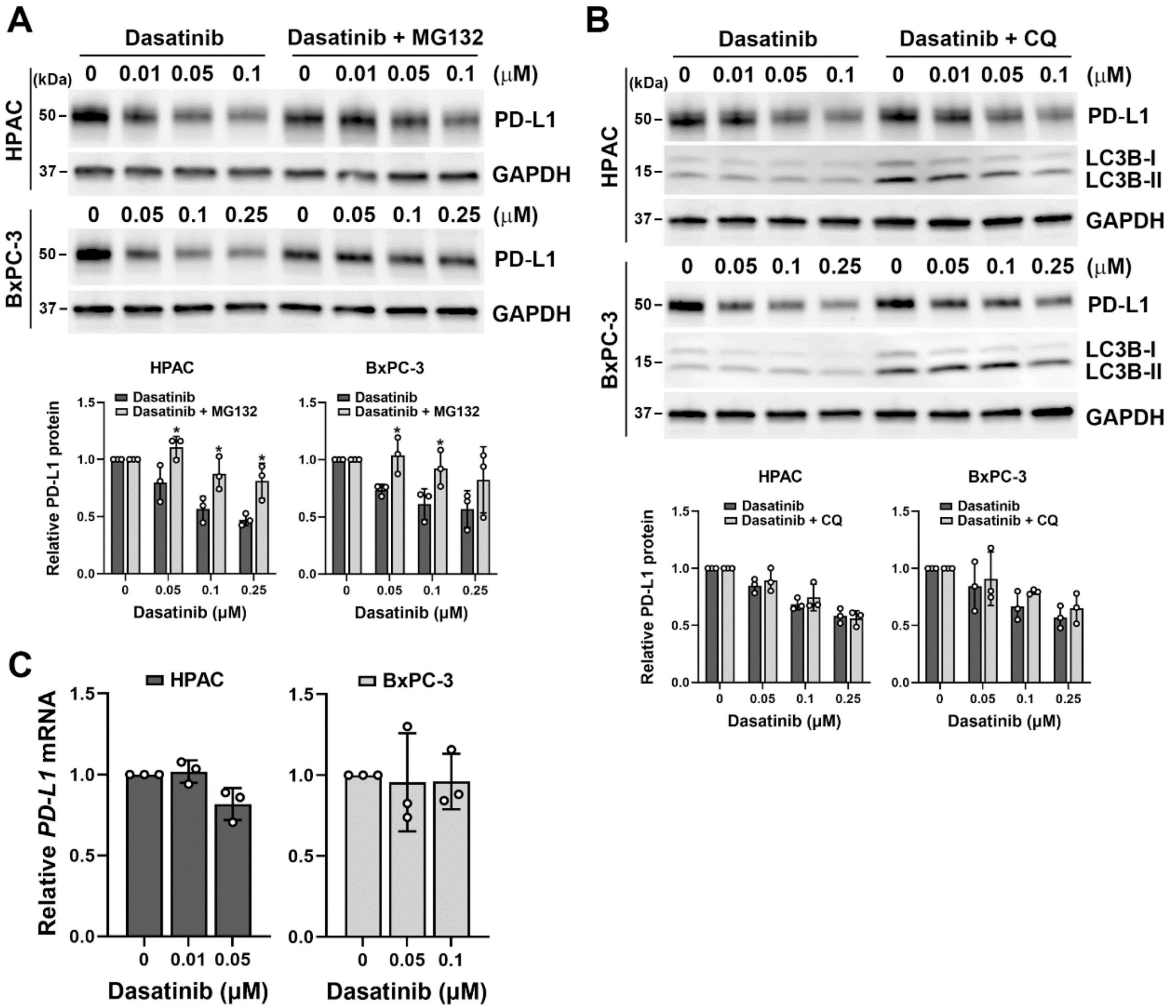
** Dasatinib promotes the proteasomal degradation of the PD-L1 protein.** (**A**) HPAC and BxPC-3 cells were treated with the indicated concentrations of dasatinib with or without 1 μM MG132 for 24 h. A Western blot analysis was performed to determine PD-L1 protein expression. Data from three independent experiments were quantified and plotted, and *p < 0.05 indicates statistical significance between treatments with and without MG132. (**B**) HPAC and BxPC-3 cells were treated with the indicated concentrations of dasatinib with or without 15 μM chloroquine (CQ) for 24 h. A Western blot analysis was performed to determine PD-L1 protein expression. Data from three independent experiments were quantified and plotted. (**C**) HPAC and BxPC-3 cells were treated with the indicated concentrations of dasatinib for 24 h. A real-time qPCR was performed to determine *PD-L1* mRNA expression.

Since dasatinib effectively reduced cell viability in PD-L1-positive/high HPAC and BxPC-3 cells (**Fig. 2B**), we investigated whether dasatinib also affects PD-L1 expression. Notably, dasatinib inhibited PD-L1 protein expression, and this effect was diminished by the proteasome inhibitor, MG-132 (**Fig. 4A**), but not by the lysosome inhibitor chloroquine (**Fig. 4B**). Additionally, dasatinib did not significantly alter *PD-L1* mRNA levels (**Fig. 4C**). These results suggest that dasatinib reduces PD-L1 expression by promoting its proteasomal degradation. Given that cancer cells use PD-L1 to evade the immune system, drugs that downregulate PD-L1 expression are believed to enhance the effectiveness of cancer immunotherapy 30-32. Furthermore, PD-L1-positive PDAC patients have significantly worse prognoses compared to PD-L1-negative patients 33. Therefore, we propose that dasatinib could be a promising treatment for PD-L1-high PDAC patients when combined with ICIs.

## Conclusions

Our study reveals a novel mechanism by which dasatinib downregulates PD-L1 expression in PDAC cells via a proteasome-dependent pathway. The correlation between PD-L1 expression and dasatinib sensitivity suggests that PD-L1 could serve as a biomarker for predicting the response to dasatinib treatment. While previous clinical trials showed limited efficacy of dasatinib in PDAC, its ability to modulate PD-L1 expression opens new avenues for combination therapies, particularly with ICIs. Future studies should focus on validating these findings in *in vivo* models and clinical settings to assess the potential of dasatinib in overcoming PDAC resistance to immunotherapy.

## Figures and Tables

**Table 1:** Correlations between the drug activity of selected TKIs and *PD-L1* mRNA expression in PDAC cell lines. Raw data were obtained from the CellMinerCDB website.

Drug name	Targets	Pearson's correlation coefficient	*P* Value
Dasatinib	SRC, ABL1, KIT	0.584	0.00549
Masitinib	KIT, PDGFRA/B	0.461	0.0406
OSI-930	KIT, VEGFR2	0.31	0.226
Tandutinib	KIT, VEGFR3	0.292	0.199
Saracatinib	SRC, ABL1	0.268	0.253
Ki8751	KIT, VEGFR2, PDGFRA	0.207	0.382
Lenvatinib	KIT, VEGFR, PDGFRA/B	0.15	0.518
Imatinib	BCR-ABL1, KIT	0.04	0.858
Bosutinib	SRC, ABL1	-0.098	0.672
KX2-391	SRC	-0.134	0.586
Sunitinib	KIT, VEGFR, PDGFRA/B	-0.154	0.504
Nilotinib	ABL1, BCR, KIT	-0.154	0.505
Axitinib	KIT, VEGFR, PDGFRA/B	-0.175	0.46
